# The Seven Ages of Man

**Published:** 1888-11-17

**Authors:** 


### The Doctor's Pulpit.

### THE SEVEN AGES OF MAN.

### VII.—"Sans Everything."

"Last scene of all,
That ends this strange eventful history,
Is second childishness, and mere oblivion;
Sans teeth, sans eyes, sans taste, sans everything."

This is a winding-up of the business of life; a closing of the battle. There are two causes of retirement from business; two ways of ending a battle. A man retires from business when he has been sufficiently successful; he also retires when he has failed and become bankrupt. A warrior closes the battle when he is victorious; he closes it, too, when he is finally conscious of defeat. It does not require a very powerful effort of the imagination to project one's thoughts forward thirty, forty, or even fifty years. In that time most people who are old enough to be interested in the reading of these non-theological sermons will be either beyond, or very close to, the last gateway on the journey that leads out of this mortal life. Will it be victory, or will it be defeat? The question is worth asking. It is not only worth asking, but, as Carlyle would say, it is, properly speaking, the question of questions.

Few men are indifferent to success. Some are so eager for it that they cannot bear the want of it, even in the smallest affairs. Everybody knows the man who always gets angry when he loses a game at whist. The woman is an everyday acquaintance, whose happiness is completely destroyed unless she can have a nobler name, a larger house, costlier furniture, more distinguished guests, than any of her neighbours. The costermonger who has the biggest and handsomest donkey, the miller who has the fattest sow, the master of foxhounds who has the finest pack and the most gallant "field" in the county—all these show their anxiety for victory, their impatience of anything like defeat. But such small circumstances are as the mere drill before the decisive battle; the preliminary trainings and canters before the final race. The whole of life is the real race every man has to run; the great and ultimate battle which every man has to fight. This won, all is won. This lost, there is hope no more.

Shakespeare is an authority among English-speaking peoples. He is probably too much of an authority; much more, indeed, than he himself ever suspected he would be. His easy breadth, his large toleration, his unvarying common sense, all commend him to the let-well-alone Englishman. It is of more than ordinary interest to hear his verdict on life as a whole. The verdict is not delivered with sighs and solemnity, but rather with a kind of ghost-like joke: as if a grinning skeleton should peer at us from the land of shadows and say "it is all over for me now, and will soon be over for you; but it is of no importance." It is in fact a plain confession of defeat. The "last scene ends this strange, eventful history." That scene is second childishness and oblivion. It is not only a scene of physical paralysis and decay, but a closing scene. Not only are teeth gone, taste gone, and eyes gone, but everything is gone. This is the very last act of the play, and then the curtain falls.

The average man, finding certain questions difficult, leaves them to specialists. He forgets that the specialist is merely a traveller who forsakes the broad highway of life in order to devote his exclusive attention to the cultivation of one small field. The fences and the gates, the soil and the drainage, the weeds and the crops peculiar to that field he will doubtless be more familiar with than the traveller who keeps to the highway; but he who walks steadfastly along the highway with open eyes and candid mind, will know infinitely more of the whole country, its capacity, and its beauty, than he who devotes himself exclusively to his one field. There are some questions which the world cannot afford to leave to specialists. No man should leave his politics to political leaders, or his religion to priests. These are questions which every one should at least attempt to settle for himself. Similarly, every intelligent person, whether man or woman, is bound by the fact of his intelligence to look at life as a whole, to consider the nature of the battle he has to fight, and to decide whether he will contend for victory or yield to defeat.

But what is victory, and what is defeat in this life of man? It is plain that physical victory is impossible. The body must and will be vanquished. Some parts of it are transformed and practically annihilated almost at once after death. Other parts, the bones for example, resist the decomposing ravages of time for years, and even centuries. But they, too, at last yield to the inevitable, and return to the companionship of their primitive elements. The victory of the physical man is, therefore, a thing practically impossible. It is, as Hume would say, contrary to experience. We are not now denying certain reputed miraculous exceptions to this rule. We are simply affirming the rule, which nobody will deny.

It must be admitted that a final defeat in life's conflict is not what the average man would prefer. No man likes to plough without reaping also; to work all the week without Saturday's wages; to enter upon a just campaign only to be finally driven into the sea. There is in all of us a deep sense that effort demands recompense, that high, noble, and persistent effort demands corresponding recompense. It is, moreover, consistent with experience that recompense follows effort, that the nature of the recompense usually corresponds to the nature of the effort. If this be so in every detail of life, but not so when life is taken as a whole, then not only have we a principle contrary to all experience established, but a principle equally contrary to the most fundamental instincts of the human mind.

The man who takes a large view of life, and after such survey deliberately chooses the highest virtue, by that resolution at once closes up many of the small gateways of success. He ensures his own defeat in a vast number of the minor battles of life. Is he to lose all these, and then at the end to be overwhelmed also in a final defeat? It seems to the present writer, notwithstanding all that such opposite people as Comtists and certain physiologists may say, that the logical issue of such a doctrine is the inevitable destruction of morals. Either man is a breath of God, and made for permanent existence, or he is a brute beast. If he be a brute beast, and must of necessity die the death of such a beast, he will be well advised to study the habits and live the life of a brute beast. Intellect and morals are distinct disadvantages on such a theory. They fill us with misery, and with a consciousness of misery which is worse than the misery itself. The present writer, though a physiologist and a student of Darwin, does not accept the verdict of Shakespeare as final. Probably, indeed, Shakespeare himself merely let the curtain drop at that point in the play because neither he nor his audience would care to go any further in thought under such circumstances. Physiology throws no light on these mysteries: it speaks neither for nor against immortality. Practical guidance is to be sought in other regions of human thought and activity.

